# Autoimmunity to HSP60 during diet induced obesity in mice

**DOI:** 10.1038/ijo.2016.216

**Published:** 2016-12-20

**Authors:** M E Şelli, G Wick, D C Wraith, A C Newby

**Affiliations:** 1 School of Clinical Sciences and Bristol Heart Institute, Bristol Royal Infirmary, University of Bristol, Bristol, UK; 2 Laboratory of Autoimmunity, Division for Experimental Pathology and Immunology, Biocenter Innsbruck Medical University, Innsbruck, Austria; 3 School of Cellular and Molecular Medicine, University of Bristol, Bristol, UK

## Abstract

Adaptive immunity has been implicated in adipose tissue inflammation, obesity and its adverse metabolic consequences. No obesity-related autoantigen has yet been identified, although heat shock protein 60 (HSP60) has been implicated in other autoimmune diseases. We investigated whether feeding a high-fat diet to C57BL/6J mice would cause autoimmunity to HSP60 and whether immunomodulation with peptides from HSP60 would reverse the resulting obesity or metabolic dysfunction. Obese mice had higher circulating levels of HSP60 associated with increased T-lymphocyte proliferation responses and the emergence of circulating IgG1 and IgG2c antibody levels against HSP60. Treatment with escalating doses of a mixture of three proven immunomodulatory HSP60 peptides did not reduce weight but completely reversed the increase in VLDL/LDL levels and partially reversed the glucose intolerance in obese mice. Obese mice mount an autoimmune response to HSP60, which partly underlies the resulting metabolic disturbances.

## Introduction

Obesity is increasing worldwide, accompanied by rising levels of type-2 diabetes and the metabolic syndrome, fatty liver disease, breast and colon cancer, musculoskeletal disorders and cardiovascular diseases, including atherosclerosis and stroke.^1, 2^ Many harmful effects of obesity have been attributed to adipose tissue (AT) inflammation,^3^ with both innate and adaptive immunity implicated.^4^ The evidence that T lymphocytes contribute to AT inflammation includes: (1) T cells accumulate in AT even before macrophages.^5, 6, 7^ (2) Restricted *V*_α_ repertoires imply antigen-specific clonal expansion.^8^ (3) Deletion of MHC Class II molecules globally or on macrophages reduces obesity, insulin resistance and AT inflammation.^9, 10^ (4) Conversely, enhancement of antigen-presenting cell function favours AT inflammation and promotes insulin resistance.^11^ This evidence suggests an autoimmune component in obesity but no culprit autoantigens have so far been identified.

HSP60 is an evolutionarily conserved mitochondrial chaperonin that can translocate to the cytosol and cell membrane and be released into the circulation under conditions of stress.^12^ HSP60 has been associated with the autoimmune component of several inflammatory diseases, including atherosclerosis.^12^ More recently, release of HSP60 from AT was demonstrated as well as its ability to cause insulin resistance and pro-inflammatory cytokine (TNF-α, IL-6 and IL-8) release by adipocytes.^13^ Also, circulating HSP60 levels were found higher in obese individuals than lean controls.^13^ All these observations make HSP60 a candidate autoantigen in obesity, although this has not yet been demonstrated. We therefore investigated whether high-fat diet (HFD) feeding gives rise to autoimmunity against HSP60 in mice and whether immunomodulation with HSP60-specific peptides can reduce obesity or the related metabolic impairment.

## Materials and methods

More detail is given in the Supplementary Methods file available at the International Journal of Obesity's website. Briefly: C57BL/6J mice (6 weeks old) purchased from Charles River Laboratories (Margate, UK) were fed normal chow (ND) or a HFD supplemented with 21% lard and 0.15% cholesterol (Special Diets Services, Witham, Essex, UK) for 16–20 weeks to induce obesity. For peptide treatment, 6-week-old mice were pre-dosed subcutaneously with HSP60 peptides (GL Biochem, Shanghai, China) starting at 0.1 μg per mouse. The dose was increased 10-fold every week up to 100 μg per mouse, which was given weekly three more times, then every 2 weeks until the end of study. HFD was started at 11 weeks of age (after the third top dose) and lasted for 20 weeks^14^ when killed by cervical dislocation under Home Office Licence 70/22957. The Guide for the care and use of laboratory animals, Eighth edition (2011) (http://grants.nih.gov/grants/olaw/guide-for-the-care-and-use-of-laboratory-animals.pdf) was followed. Procedures were carried out under Home Office Licences 30/3064 and 70/22957. All animals survived until killed and were included in the analysis.

After killing, epididymal fat pads were collected, weighed and the stromal vascular fraction (SVF) was isolated by collagenase digestion. For analysis of macrophage populations, 1 million SVF cells were examined by flow cytometry analysis using antibodies against CD11b, F4/80, CD11c and CD206. T-cell populations were analysed using antibodies against CD45, CD3ɛ, CD4, CD25 and FoxP3.

Serum HSP60 levels were measured with a mouse HSP60 ELISA (NeoScientific, Cambridge, MA, USA). Serum anti-HSP60 antibody levels were measured with a custom-made ELISA using recombinant, endotoxin-depleted murine HSP60 protein (Enzo Life Sciences, Farmingdale, NY, USA) bound to Nunc Immuno MaxiSorp 96-well plates. For the HSP60 reactive T-cell proliferation assay, total cell pellets from homogenised spleens were pulsed with ^3^H-thymidine for 18 h after pre-treating with buffer control, recombinant HSP60 or peptides.

Glucose tolerance tests were performed after 16 weeks of ND or HFD. After 6 h fast, 2 g kg^−1^ body weight of glucose was injected intraperitoneally and glucose concentration in blood from tail snips was measured 0, 15, 30, 60 and 90 min later. Insulin tolerance test was conducted one week later. After 4 h fast, rapid acting human insulin (NovoRapid; Novo Nordisk A/S, Bagsvaerd, Denmark) was injected intraperitoneally to give a final dose of 1 U kg^−1^ body weight. Blood glucose was measured at the same time points. Mouse Ultrasensitive Insulin ELISA kit (Alpco, Salem, NH, USA) was used to determine fasting insulin levels.

For normally distributed variables (Kolmogorov–Smironov test), a two-tailed, unpaired Student's *t*-test was used to determine significant differences between 2 means. For multiple comparisons, a one-way ANOVA or two-way ANOVA was performed, as appropriate, followed by a Bonferroni correction. Differences were considered significant if *P*<0.05.

## Results

### Increased circulating HSP60 levels, antibodies against HSP60, and T-cell responses to HSP60 in HFD-fed C57BL/6J mice

**Figure 1:**
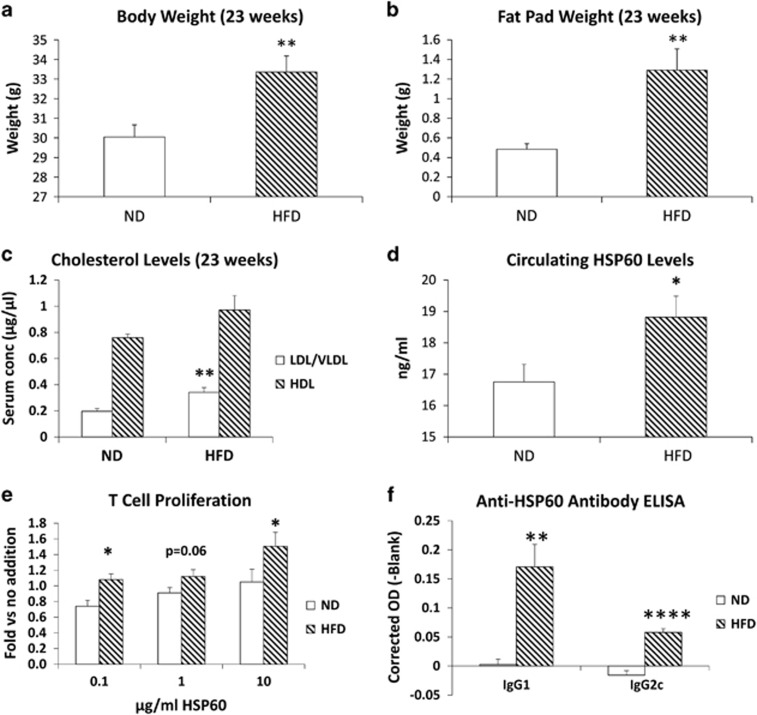
Effect of HFD on weight gain, metabolism and autoimmunity to HSP60. Groups of mice (*n*=7) were fed HFD or ND for 16 weeks. (**a**) Body weights (*P*=0.007) and (**b**) epididymal fat pad weights (*P*=0.004) were significantly increased by HFD. (**c**) Circulating LDL/VLDL levels were also significantly increased (*P*=0.005). (**d**) Mouse HSP60 levels measured in serum samples by ELISA were significantly higher in HFD compared with ND fed mice (*P*=0.039; *n*=19 each). (**e**) T-cell proliferation (expressed relative to the medium alone negative control) increased significantly in HFD compared with ND splenoctes when stimulated by 0.1 μg ml^−1^ (*P*=0.013) or 10 μg ml^−1^ (*P*=0.038) of recombinant mouse HSP60. (**f**) Both anti-HSP60 IgG1 and IgG2c circulating antibody levels were significantly elevated by HFD (*P*_max_=0.008 for IgG1; *P*_max_=0.00009 for IgG2c).

C57BL/6J mice fed HFD for 16 weeks were 3 g heavier than ND mice (Figure 1a) and their epididymal fat pads were almost three times heavier (Figure 1b). Plasma levels of LDL/VLDL were significantly increased (74%) by HFD, although HDL levels were unaltered (Figure 1c). HFD significantly increased circulating HSP60 levels by 12% (Figure 1d), suggesting that obesity is a stress factor capable of stimulating HSP60 release.

As evidence for an autoimmune reaction to HSP60, recombinant HSP60 increased splenic T-lymphocyte proliferation relative to buffer controls by 20–40% more in HFD than ND mice. This increase was significant at 0.1 and 10 μg ml^−1^ and it was at borderline significance (*P*=0.063) at 1.0 μg ml^−1^ concentration (Figure 1e). The response to HSP60 was much weaker than the positive control ConA (13-fold, not shown), not surprisingly because the mice were not hyper-immunized. Circulating anti-HSP60 levels were undetectable in ND mice but HFD significantly elevated both anti-HSP60 IgG1, which is mainly dependent on Thelper2 (Th2) lymphocytes, and IgG2c, which is driven by Th1 lymphocytes^15^ (Figure 1f). Differences between HFD and ND mice were highly significant above 1/16 dilution; much lower than expected for hyper-immune serum (typically >1/1000). Clearly, short periods of obesity triggered autoimmunity against murine HSP60, albeit at low level.

### Effect of HSP60 peptide treatment on obesity and related metabolic dysfunction

To investigate the impact of HSP60 autoimmunity, we used subcutaneous HSP60 peptide treatment with a combination of equal concentrations of three peptides known to cause favourable immunomodulation in models of autoimmune diabetes or atherosclerosis. These were vlgggcallrcipaldslkpaned,^16^ aelkkqskpvt^17^ and dgealstlvlnrlkvg.^18^ Each of these peptides had good solubility in water (results not shown). We used a dose escalation protocol that has proved safe and effective against experimental autoimmune encephalomyelitis.^14^

**Figure 2:**
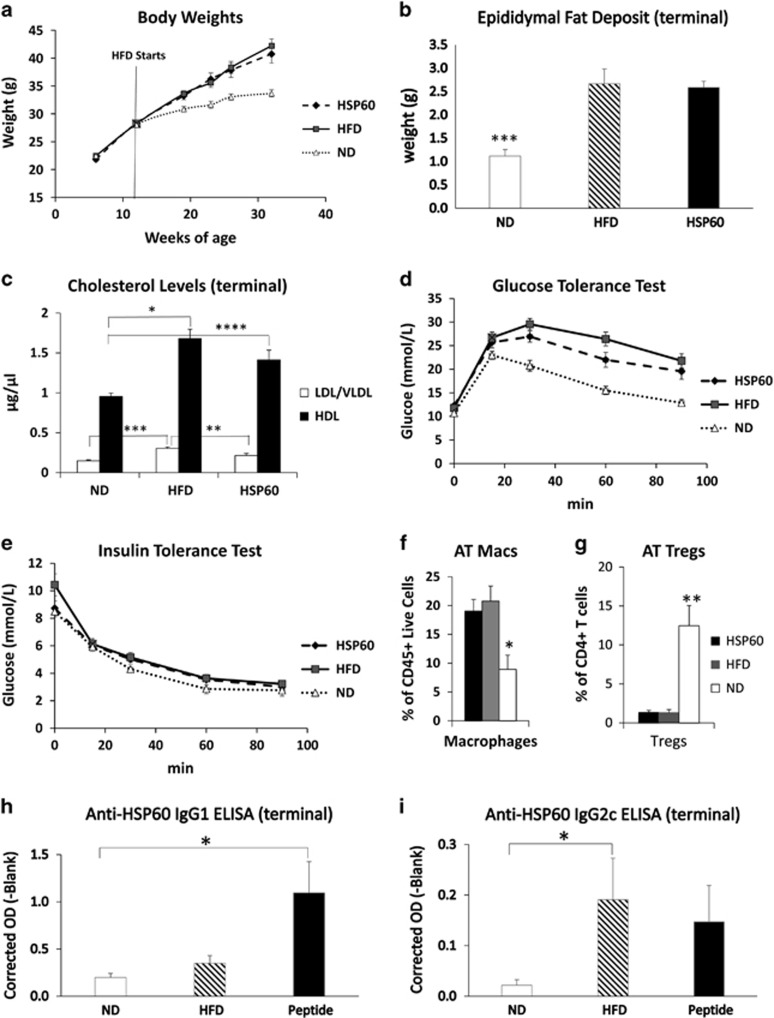
Effect of HSP60 peptide treatment on obesity, metabolic dysfunction and autoimmunity to HSP60. (**a**) Mice (*n*=14) given HFD or HFD with HSP60 peptides increased weight to the same extent relative to ND (*P*=0.000002 for HFD, *P*=0.00045 for HSP60). (**b**) Epididymal fat pad weights were similarly doubled by HFD or HFD with HSP60 peptides (*P*=0.0012 for HFD, *P*=0.00009 for HSP60). (**c**) HFD increased LDL/VLDL (*P*=0.0003) and HDL levels (*P*=0.00045) and the HSP60 treatment significantly normalised LDL/VLDL levels (*P*=0.0088) but not HDL levels. (**d**) HFD significantly impaired glucose tolerance (*P*=3.4 × 10^−18^, two-way ANOVA) and the impairment was partially but significantly reversed by HSP60 peptide treatment (*P*=0.0079). In a subgroup (*n*=7 each): (**e**) no differences were found in insulin tolerance between the three groups. (**f**) From flow cytometry, epididymal stromal vascular fraction CD45^+^CD11b^+^F4/80^+^ macrophages were increased (*P*=0.013) and (**g**) CD45^+^CD3^+^CD4^+^CD25^+^Foxp3^+^ regulatory T cells were decreased significantly (*P*=0.003) with HFD or HFD plus HSP60 peptides. (**h**) There was an increase in the level of anti-HSP60 IgG1 antibodies observed in both HFD groups, but only the increase in the peptide treatment group was significant (*P*=0.022). (**i**) Anti-HSP60 IgG2c antibodies were increased in HFD mice, but this was significant only in the HFD alone group (*P*=0.048 for 1/16 dilution).

HSP60 peptide treatment did not reverse HFD-induced weight gain (Figure 2a) or the increase in epididymal fat mass (Figure 2b), which were greater in this experiment owing to the longer period of HFD feeding. However, peptide treatment reversed the significantly increased circulating LDL/VLDL levels in HFD mice (Figure 2c).

Glucose tolerance was significantly impaired in HFD compared to ND mice. HSP60 peptide treatment rendered HFD mice significantly more glucose tolerant, although not as tolerant as ND mice (Figure 2d). Fasting insulin levels were elevated to the same degree relative to ND (0.64±0.05 ng ml^−1^) by HFD, irrespective of peptide treatment (1.01±0.22 and 1.18±0.22 ng ml^−1^) and could not account for the improved glucose tolerance. Insulin sensitivity, measured in an insulin tolerance test, was also similar in ND, HFD and peptide treatment groups (Figure 2e).

To investigate whether HSP60 peptide treatment suppressed obesity-associated inflammation, we subjected SVF cells to flow cytometry. We recorded a significant increase in macrophages (Figure 2f), no change in total CD4^+^ or CD4^−^ T lymphocytes (not shown) and a decrease in Treg cells (Figure 2g) in HFD compared with ND mice but there was no effect of HSP60 peptide treatment (Figures 2f and g). We also did not observe any difference in splenic T-cell proliferation responses to recombinant HSP60 in HFD-fed mice with and without HSP60 peptide treatment. Nor did we find any difference in the levels of IL-2, IL-4, IL-6, IL-10, IL-17, IFN-γ or TNFα released spontaneously or in response to HSP60 challenge (results not shown). However, HSP60 peptide treatment increased the titre of anti-HSP60 IgG1 (Figure 2h) but not IgG2c antibodies (Figure 2i), which implies an enhancement of Th2-driven antibody production.

## Discussion

An autoimmune component of obesity has been postulated^8, 9^ (see Introduction) but our study is the first to identify a contributing autoantigen. Obese mice clearly mounted a weak autoimmune response to HSP60 at both the T- and B-cell levels after a short period of HFD. Consistent with this, lean humans have antibodies to HSP60, presumably arising from other stressors,^12^ but the level of autoantibodies is increased by obesity that is clearly much more prolonged than in our mice.^13^ Moreover, we could reverse the hypercholesterolaemic effect of HFD and partially improve glucose tolerance with immunomodulatory HSP60 peptide treatment, implying that autoimmunity to HSP60 contributed to the metabolic disturbances caused by obesity, despite weight not being reduced. A similar decoupling of obesity and metabolic disturbances has been noted in other studies when suppressing adaptive immunity, for example in RAG-1-deficient mice, which lack mature T and B lymphocytes.^8^ Given observations that LDL production is increased by obesity^19^ and that HSP60 autoimmunity contributes to atherosclerosis,^12^ the reduction in LDL levels achieved by HSP60 peptide treatment is of interest. A recent study showing that regulatory T cells can reduce the levels of blood cholesterol in low density lipoprotein deleted mice might provide a mechanisms for our results,^20^ but this requires further investigation.

HFD increased AT macrophages and decreased Treg as previously reported by others (reviewed by Lolmede *et al.*^4^) but we did not see any changes with HSP60 peptide treatment. Neither did we find any influence of peptide-induced splenic T-cell proliferation or cytokine release. We did, however, see a switch towards Th2-driven IgG1 production, which implies that HSP60 peptide treatment caused immunomodulation rather than immune tolerization, which will require more extensive investigation. In summary, we showed for the first time that low-level autoimmunity to HSP60 contributes to, and HSP60 peptide treatment partially reverses, metabolic disturbances in a murine obesity model.
