# Supplementary material for: Autoimmunity to HSP60 during diet induced obesity in mice
Source: Int J Obes (Lond). 2016 Dec 20;41(2):348–51. doi: 10.1038/ijo.2016.216 (PMC5300117; doi:10.1038/ijo.2016.216)
Supplement: Supplementary Methods [file ijo2016216x1.docx]

**Supplementary methods**

*Mouse feeding and treatment with immunomodulatory peptides.* Male C57BL/6J mice (6 weeks old) obtained and housed at Charles River Laboratories were fed normal chow (ND) or a high fat diet (HFD) supplemented with 21% lard and 0.15% cholesterol (Special Diets Services) for 17 weeks to induce obesity. HSP60 peptides were ordered from GL Biochem (Shanghai) with a minimum 85% purity. The lyophilised peptides were dissolved in DMSO and diluted in PBS at 20 mg/mL determined using Pierce BCA Protein assay kit (ThermoFisher Scientific). Serial dilutions in PBS were conducted and the protein concentration was re-measured. A linear decline in concentration with dilution was taken to indicate good solubility in water. For HSP60 peptide treatment, C57BL/6J mice were ordered from Charles River at 5 weeks of age and divided into 3 groups (14 mice per group from 2, 3 or 4 different litters, respectively) as follows: 1) ND control, 2) HFD, 3) HFD plus HSP60 peptide treatment. After 1 week for acclimatisation, mice were pre-dosed with HSP60 peptides starting at 0.1 µg/mouse in phosphate buffered saline (PBS). The dose was increased 10 fold every week until it reached 100 µg/mouse. The top dose was given weekly 3 more times, then every two weeks until the end of study^1^. HFD was started at 11 weeks of age (after the third top dose) and lasted for 20 weeks. Based on previous experience^1^ groups of 7 animals were chosen for the initial studies. However, given the weak autoimmune reaction obtained sample size was arbitrarily increased to n=14 for the peptide treatments. Where sufficient plasma was available, HSP60 levels were measured in both groups of mice (n=19 from a possible n=21). All animals survived until culled and were included in the analysis. All mice were coded so that all the laboratory analyses could be carried out blinded as to treatment.

*Measuring serum HSP60 and antibodies to HSP60.* Bloods were collected from the abdominal aorta under terminal anaesthesia and kept at room temperature for 30 min, then at least 2 hours on ice to allow blood sufficient time to clot. Serum samples were then separated by 15 min centrifugation at 8500 x g. To determine serum HSP60 levels, an ELISA based quantitative mouse HSP60 kit (NeoScientific) was used according to the manufacturer’s instructions. To measure antibodies to HSP60, Nunc Immuno MaxiSorp 96 well flat bottom plates were coated with 100μL of 4 µg/ml recombinant murine HSP60 protein (Enzo Life Sciences) in 0.05M carbonate-bicarbonate buffer (pH 9.0) and incubated overnight at 4°C. The plates were then washed four times with 0.05% (v/v) Tween-20 in PBS (PBST). Non-specific binding sites were blocked by adding 100μL 2% BSA (Sigma-Aldrich) then 100 μL of serum samples diluted 1:4, 1:8 and 1:16 in 1% BSA 0.01% Tween-20 in PBS was added and incubated overnight at 4°C. Bound antibodies were detected with 1:1000 of goat anti-mouse IgG1 or IgG2c secondary antibodies conjugated to alkaline phosphatase (Abcam) followed by washing with PBST. 100 μl of the p-Nitrophenyl Phosphate Liquid Substrate System (Sigma-Aldrich) was then added and colour (405 nm) was developed over 1h.

*Serum levels of lipoproteins and measurement of atherosclerosis.* Serum was obtained as described above. The HDL and LDL/VLDL Cholesterol Assay Kit (Abcam) was used to quantify the levels of cholesterol. Briefly, HDL was first separated from LDL/VLDL (or serum used unseparated to measure the total cholesterol), then cholesterol oxidase was added to the samples which recognizes free cholesterol and produces products reacting with probe to generate colour. The intensity of colour was then measured using LT-5000MS ELISA Reader (Labtech) at a wavelength of 570 nm. To investigate atherosclerotic plaque development, 5 µm histological sections were taken from the aortic sinus at the level of the valves as described by Paigen and colleagues^2^. Sections were stained with haematoxylin and eosin and the area of plaque was quantified from micrographs using Image Pro (DataCell).

*Harvesting epididimal fat pads and production of SVF cells.* After euthanasia by cervical dislocation under Home Office Licence 70/22957, epididymal fat pads were collected, weighed and were used prepare SVF, as described previously^3^. Briefly, fat pads were placed into phosphate buffered saline (PBS; 2.7 mM KCl, 1.5 mM KH_2_PO_4_, 136.9 mM NaCl, 8.1 mM Na_2_HPO_4_.7H_2_O) and minced using a scalpel and forceps on ice. A bacterial lipopolysaccharide (LPS)-depleted collagenase cocktail (Liberase TM Grade, Roche) (0.03 mg/ml) and 50 U/ml DNase I (Sigma Aldrich) was added into minced samples and incubated for 30 min on an orbital shaker at 37°C. Digested samples were filtered using 70 nm nylon cell strainer followed by centrifugation at 400xg for 5 min. The pellet was re-suspended in 0.5 ml of erythrocyte lysis buffer (Invitrogen) and incubated at room temperature for 5 min. Finally, erythrocyte free samples were centrifuged at 400xg for another 5 min and the pelleted cells were used as SVF.

*Flow cytometry.* Approximately 1 million SVF or peritoneal cells were washed with PBS and incubated with 0.5 µl Fixable Viability Dye eFluor 780 (eBioscience) in 0.5 ml PBS for 30 min on ice. Cells were re-suspended in 100 µl staining buffer (0.5% FCS, 2 mM EDTA, 25 mM HEPES in PBS) after washing with the same buffer. The Fc receptors present on cells were blocked by addition of 2 µl Fc block (TruStain fcX; anti-mouse CD16/32, Biolegend 101319) followed by a 5 min incubation on ice to prevent unspecific background staining. Then, the cells were incubated on ice for 30 min in the dark with an appropriate amount of fluorescently labelled anti-mouse antibodies as detailed below. Between two washing steps with 2 ml staining buffer, 1 µl streptoavidin was added to the corresponding tubes followed by 15 min incubation on ice. Finally, the pelleted cells were re-suspended in 0.3 ml fixation buffer (1% paraformaldehyde in staining buffer) and kept in the dark at 4ºC until flow cytometry analysis. Macrophage populations were analysed using combinations of the following mouse monoclonal antibodies. 2.5 µg/ml rat CD11b labelled with pacific blue (Biolegend 101223), 5 µg/ml hamster CD11c labelled with Alexa Fluor 700 (Biolegend 117319), 4 µg/ml rat CD206 labelled with phycoerythrin (Biolegend 141705), 2 µg/ml rat F4/80 labelled with biotin (AbD Serotec MCA497B) followed by 2 µg/ml streptavidin labelled with Alexa Fluor 488 (Invitrogen S32354). T-cell populations were analysed using combinations of the following mouse monoclonal antibodies (all from Biolegend): CD45 clone 30-F11 Brilliant Violet 510, 5.0 µg/ml; CD3ε clone 145-2C11 PerCP-Cy.5.5, 2.5 µg/ml; CD4 clone RM4-5 Alexa Fluor 700, 2.5 µg/ml; CD25 clone 3C7 Phycoerythrin, 10.0 µg/ml; FoxP3 clone MF-14 Alexa Fluor 488, 2.5 µg/ml; F4/80 clone BM8 PE-Cy7, 2.5 µg/ml; CD11b clone M1/70 Brilliant Violet 421, 2.5 µg/ml; CD11c clone N418 Brilliant Violet 785, 50.0 µl/ml; CD206 clone C068C2 APC, 5.0 µg/ml.

*Isolation of murine splenocytes.* Spleens were collected into RPMI 1640 (ThermoFisher Scientific) media supplemented with 5% FCS, 2 mM L-Glutamine, 100 IU/ml Penicillin and 100 µg/ml Streptomycin (will be referred as complete RPMI) or X-VIVO 15 (Lonza) media supplemented with 2 mM L-Glutamine, 100 IU/ml Penicillin and 100 µg/ml Streptomycin, then homogenized using a 5 ml syringe plunger and a sterile petri dish. To prepare splenocytes^4^, the homogenate was filtered into 50 ml falcon tubes using 40 nm cell strainers. The cell suspension was then completed to 40 ml with PBS followed by a centrifugation at 300 x g for 5 min at 4 °C. The pellet was re-suspended in 2 ml of red blood cell lysis buffer (Sigma Aldrich) and incubated for 10 min at room temperature. Erythrocyte depleted cells were then washed with 40 ml PBS followed by 5 min centrifugation at 300 x g. The pellet was re-suspended in 2 ml PBS (if cells were going to be stained for flow cytometry) or media (if cells were going to be cultured). Finally, cells were counted using a haemocytometer.

*HSP60 reactive T cell proliferation assay*. As optimized from Anderton and Wraith^5^, erythrocyte depleted splenocytes were cultured in 96 well plates (5x10^5^ cells/well) for 72 hours at 37 C° at 5% CO_2_ in the presence of 0, 0.1, 1.0 and 10.0 µg/ml of recombinant HSP60 protein (Enzo Life Sciences) or Concanavalin A (2 µg/ml). Cells were then pulsed with 0.5 µCi/well of ^3^H-thymidine for 18 hours before harvesting onto glass fibre filters (Cox Scientific) and placing into a 1450 Microbeta Liquid Scintillation Counter (Perkin Elmer). For cytokine analysis the flow cytometry based BD Cytometric Bead Array (CBA) Mouse Th1/Th2/Th17 Cytokine Kit (Cat No: 560485) was used according to the manufacturer's instructions.

*Glucose and insulin tolerance tests.* Glucose tolerance tests were performed after 16 weeks of ND or HFD, as described previously^6^. Food was withdrawn 6 hours prior to the test. The mice were weighed and the fasting blood glucose levels were measured using Accu-Check Aviva glucometer (Roche) in tail vein blood to get the value for 0 time point. Tail snipping was used to obtain blood. Before snips, Emla Cream 5% (AstraZeneca) was applied to the tail ends for local anaesthesia to reduce pain. 20% glucose solution was prepared in PBS. The amount of glucose solution to be injected was calculated by multiplying the body weight (g) by 10 µl to adjust the glucose dose for 2 g/kg body weight. For example, 400 µl of 20% glucose solution was injected into the peritoneal where the mouse weighed 40 g. Blood glucose was measured at 15, 30, 60, and 90 minutes after the glucose injection.

Insulin tolerance tests were conducted one week later using a previously described protocol^6^. Animals were fasted 4 hours and then were injected with rapid acting human insulin (NovoRapid; Novo Nordisk A/S) at 0.1 U/ml in PBS. After measuring the fasting glucose level, 10 x body weight (g) µl insulin was injected intraperitoneally to give a final dose of 1 U/kg body weight. Blood glucose was again measured at 15, 30, 60, and 90 minutes after the insulin injection^6^. Mouse Ultrasensitive Insulin ELISA kit (Alpco) was used to determine fasting insulin levels.

**References**

1. Burton BR, Britton GJ, Fang H, Verhagen J, Smithers B, Sabatos-Peyton CA *et al.* Sequential transcriptional changes dictate safe and effective antigen-specific immunotherapy. *Nat Commun* 2014; **5:** 4741.

2. Paigen B, Morrow A, Holmes PA, Mitchell D, Williams RA. Quantitative assessment of atherosclerotic lesions in mice. *Atherosclerosis* 1987; **68**(3)**:** 231-40.

3. Zeyda M, Farmer D, Todoric J, Aszmann O, Speiser M, Györi G *et al.* Human adipose tissue macrophages are of an anti-inflammatory phenotype but capable of excessive pro-inflammatory mediator production. *Int J Obes (Lond)* 2007; **31**(9)**:** 1420-8.

4. Stagg AJ, Burke F, Hill S, Knight SC. Isolation of mouse spleen dendritic cells. *Methods Mol Med* 2001; **64:** 9-22.

5. Anderton SM, Wraith DC. Hierarchy in the ability of T cell epitopes to induce peripheral tolerance to antigens from myelin. *Eur J Immunol* 1998; **28**(4)**:** 1251-61.

6. Ayala JE, Samuel VT, Morton GJ, Obici S, Croniger CM, Shulman GI *et al.* Standard operating procedures for describing and performing metabolic tests of glucose homeostasis in mice. *Dis Model Mech* 2010; **3**(9-10)**:** 525-34.
